# A 2026 Update on Computational Approaches to the Discovery and Design of Antimicrobial Peptides

**DOI:** 10.3390/antibiotics15020203

**Published:** 2026-02-13

**Authors:** Guillermin Agüero-Chapin, Agostinho Antunes, Yovani Marrero-Ponce

**Affiliations:** 1 CIIMAR/CIMAR LA, Interdisciplinary Centre of Marine and Environmental Research, University of Porto, 4450-208 Porto, Portugal; aantunes@ciimar.up.pt; 2 Department of Biology, Faculty of Sciences, University of Porto, 4169-007 Porto, Portugal; 3 Facultad de Ingeniería, Universidad Panamericana, Ciudad de México 03920, Mexico; 4 Grupo de Medicina Molecular y Traslacional (MeM&T), Escuela de Medicina, Edificio de Especialidades Médicas, Colegio de Ciencias de la Salud (COCSA), Universidad San Francisco de Quito (USFQ), Quito 170157, Ecuador

Antimicrobial resistance (AMR) continues to stand as a critical global healthcare challenge. Current projections suggest that AMR could become the leading cause of death by 2050, potentially surpassing cancer mortality rates [1]. The urgency for novel therapeutic agents was further underscored by the 2020 pandemic, which shifted antibiotic consumption patterns and accelerated the emergence of multidrug-resistant (MDR) “superbugs” [2]. In this precarious landscape, Antimicrobial Peptides (AMPs) represent a transformative frontier. Unlike traditional antibiotics, AMPs typically target microbial membranes, a mechanism that significantly diminishes the likelihood of resistance development [3,4].

The conventional discovery of AMPs is a laboratory-intensive process often hampered by prohibitive costs and protracted timelines. This Special Issue, “A 2026 Update on Computational Approaches to the Discovery and Design of Antimicrobial Peptides”, showcases the power of in silico methodologies to accelerate the identification and optimization of these therapeutic candidates [5].

## 1. The AI Revolution and Modern Classification

Recent breakthroughs in Artificial Intelligence (AI) and Machine Learning (ML) have fundamentally changed how researchers navigate the vast chemical space of AMPs [5]. Contributions to this Special Issue present robust Deep Learning (DL) frameworks, such as the dsAMP and dsAMPGAN models, which utilize CNN-Attention-BiLSTM architectures and Generative Adversarial Networks (GANs) to predict peptide activity and synthesize novel sequences. Furthermore, the introduction of the AntiBP3 method provides an alignment-free approach to target-specific classification against Gram-positive, Gram-negative, and Gram-variable bacteria.

Innovative alignment methods also play a pivotal role. The integration of Multi-Query Similarity Search Models (MQSSMs) into the StarPep toolbox has demonstrated superior performance in identifying antiviral peptides (AVPs) while optimizing computational resources [6]. Additionally, research leveraging Geometric Deep Learning and ESMFold-predicted tertiary structures has shown that utilizing non-Euclidean distance functions allows for the creation of topologically diverse graphs that better capture the structural features essential for antiviral potency (Contributions 1–4).

## 2. Structural Insights and Molecular Mechanisms

This Special Issue explores the intricate relationship between peptide structure and membrane interaction, a cornerstone in designing effective drugs. A comparative study on RWV peptides (comprising Arginine, Tryptophan, and Valine) revealed that linear amphipathicity—segregating cationic and hydrophobic residues in the primary sequence—can yield higher antibacterial potency at shorter lengths compared to traditional helical motifs.

Targeted design is also highlighted in the development of the HRZN peptide series. By utilizing database filtering and positional analysis, researchers developed HRZN-15, which targets MDR *Acinetobacter baumannii* with rapid killing kinetics and the capacity to eradicate pre-formed biofilms. These structural advancements are further supported by high-resolution Molecular Dynamics (MD) simulations. Such simulations provided the structural determinants for the adsorption of the hybrid AMP CIDEM-501, helping to rationalize its potent activity against MDR pathogens (Contributions 5–7).

## 3. Rational Design and Multifunctionality

The research within this Special Issue also expands the functional definition of AMPs, illustrating their potential in wound healing and tissue regeneration. By employing molecular docking to analyse binding affinities with growth factor receptors (such as EGFR and VEGFR), studies on cationic peptides derived from Cecropin D demonstrate how these molecules can promote cell migration and re-epithelialization (Contribution 8).

## 4. Conclusions

The synergy between computational prediction and experimental validation presented in this Special Issue provides a powerful toolkit for the next generation of peptide therapeutics. We hope that these contributions will serve as a catalyst for future research in our collective effort to overcome antimicrobial resistance.
